# Endosidin 5 disruption of the Golgi apparatus and extracellular matrix secretion in the unicellular charophyte *Penium margaritaceum*

**DOI:** 10.1093/aob/mcad054

**Published:** 2023-04-20

**Authors:** Josephine G LoRicco, Li Kozel, Kaylee Bagdan, Ruby Epstein, David S Domozych

**Affiliations:** Department of Biology and Skidmore Microscopy Imaging Center, Skidmore College, Saratoga Springs, NY, 12866USA; Department of Biology and Skidmore Microscopy Imaging Center, Skidmore College, Saratoga Springs, NY, 12866USA; Department of Biology and Skidmore Microscopy Imaging Center, Skidmore College, Saratoga Springs, NY, 12866USA; Department of Biology and Skidmore Microscopy Imaging Center, Skidmore College, Saratoga Springs, NY, 12866USA; Department of Biology and Skidmore Microscopy Imaging Center, Skidmore College, Saratoga Springs, NY, 12866USA

**Keywords:** Charophytes, secretion, endomembrane system, Golgi apparatus, endosidin, brefeldin A, concanamycin A, tomography

## Abstract

**Background and Aims:**

Endosidins are a group of low-molecular-weight compounds, first identified by ‘chemical biology’ screening assays, that have been used to target specific components of the endomembrane system. In this study, we employed multiple microscopy-based screening techniques to elucidate the effects of endosidin 5 (ES5) on the Golgi apparatus and the secretion of extracellular matrix (ECM) components in *Penium margaritaceum*. These effects were compared with those caused by treatments with brefeldin A and concanamycin A. *Penium margaritaceum*’s extensive Golgi apparatus and endomembrane system make it an outstanding model organism for screening changes to the endomembrane system. Here we detail changes to the Golgi apparatus and secretion of ECM material caused by ES5.

**Methods:**

Changes to extracellular polymeric substance (EPS) secretion and cell wall expansion were screened using fluorescence microscopy. Confocal laser scanning microscopy and transmission electron microscopy were used to assess changes to the Golgi apparatus, the cell wall and the vesicular network. Electron tomography was also performed to detail the changes to the Golgi apparatus.

**Key Results:**

While other endosidins were able to impact EPS secretion and cell wall expansion, only ES5 completely inhibited EPS secretion and cell wall expansion over 24 h. Short treatments of ES5 resulted in displacement of the Golgi bodies from their typical linear alignment. The number of cisternae decreased per Golgi stack and *trans* face cisternae in-curled to form distinct elongate circular profiles. Longer treatment resulted in a transformation of the Golgi body to an irregular aggregate of cisternae. These alterations could be reversed by removal of ES5 and returning cells to culture.

**Conclusions:**

ES5 alters secretion of ECM material in *Penium* by affecting the Golgi apparatus and does so in a markedly different way from other endomembrane inhibitors such as brefeldin A and concanamycin A.

## INTRODUCTION

The extracellular matrix (ECM) of plants includes the cell wall, an assortment of gel-like polysaccharides/proteoglycans and hydrophobic materials that are deposited either onto or in the cell wall. The structural and functional dynamics of the ECM are profoundly important for the life of the plant cell. The cell wall plays a central role in the control of cell expansion and morphogenesis, provides a rigid edifice for physical support, comprises a signalling network as well as a structural and biochemical barrier for resistance to abiotic and biotic stressors, and contributes to absorption and transport of water and solutes (Tenhaken, 2015; Chebli and Geitmann, 2017; Zhang *et al.*, 2021; Cosgrove, 2022). Other ECM components function by providing a hydrated matrix for embryo development during seed germination (Tsai *et al.*, 2021), serving as lubricants for root growth in soils (Driouich *et al.*, 2021) or providing waterproof coatings on various tissues and organs (e.g. cuticles; Domínguez *et al.*, 2011; Niklas *et al.*, 2017; Arya *et al.*, 2021). Production of the ECM requires the coordinated actions of multiple subcellular systems that are synchronized during cell expansion and development and can rapidly modulate in response to environmental stress. The primary machinery for ECM processing (i.e. biosynthesis, packaging and transport to the cell surface, secretion and incorporation into the ECM architecture) consists of the endomembrane system (Kim and Brandizzi, 2014), cytoskeletal network (Gu and Rasmussen, 2022), specific loci of the plasma membrane (vesicle tethering complex or exocyst; Huang *et al.*, 2019a,b; Saeed *et al.*, 2019) and even the ECM itself (Bacete *et al.*, 2018). The endomembrane system is the central subcellular unit in this processing and its main components, the endoplasmic reticulum (ER), the Golgi apparatus, the *trans* Golgi network (TGN) and various types of vesicles, have been extensively studied in plants (Worden *et al.*, 2012; Drakakaki, 2015; van de Meene *et al.*, 2017; Sinclair *et al.*, 2018; Robinson, 2020; Aniento *et al.*, 2022; Nakano, 2022; Shimizu and Uemura, 2022). The Golgi apparatus is a major ‘cog’ in the ECM-processing machine. Its distinct architectural design and highly complex biosynthesis machinery are directly involved in the synthesis, packaging and transport of ECM components to the cell exterior.

Analyses of the Golgi apparatus and endomembrane system in plants have greatly benefitted from the application of diverse and novel technologies. These include glycomic analyses of isolated endomembrane components (Okekeogbu *et al.*, 2019; Wilkop *et al.*, 2019), mutation analysis (Kohorn et al, 2021), electron microscopy and tomography (Koga *et al.*, 2016; Otegui and Pennington, 2019; Weiner *et al.*, 2022), 3-D printing (Mai *et al.*, 2017) and the application of a wide array of fluorescent markers (DeVree *et al.*, 2021) including fluorescence-tagged marker proteins in transformed cell lines. Another important tool used in endomembrane system research is chemical genetics/chemical biology (Hicks and Raikhel, 2010; Norambuena and Tejos, 2017; Rodriguez-Furlan *et al.*, 2018; Ma *et al.*, 2022). Here, application of small-molecule inhibitors targeting specific endomembrane components allows for transient manipulation of the membrane trafficking processes in both a notable and reversible manner. Alterations to the endomembrane system and secretory cargoes may then be monitored and even quantified using high-resolution live-cell fluorescence light microscopy (FLM)-based imaging, and detailed ‘snapshots’ of specific stages of alteration during the treatment may be acquired using various transmission electron microscopy (TEM)-based techniques. Inhibition effects may ultimately be checked via recovery experiments and their severity can be controlled by manipulating dosage. Several chemical agents have become powerful tools for dissection of the Golgi apparatus and other endomembrane components (e.g. brefeldin A; Nebenführ *et al.*, 2002; Foissner *et al.*, 2020)).

Plants evolved from a freshwater charophycean green alga (i.e. basal streptophyte) ~500–600 million years ago (Mattox and Stewart, 1984; de Vries and Archibald, 2018; Rensing, 2018). The ECM of these algae was critical to the successful invasion of land and subsequent evolution into the great diversity of modern land plants (Sørensen *et al.*, 2011; Harholt *et al.*, 2016). Phylogenomic analyses have now clearly confirmed that the Zygnematophyceae are the sister group to embryophytes (Delwiche and Cooper, 2015; de Vries and Archibald, 2018; Puttick *et al.*, 2018; Zhou and von Schwartzenberg, 2020; Hess *et al.*, 2022). Like most land plants, most modern-day zygnematophyte taxa possess a complex ECM. This includes a cell wall with constituents that are remarkably similar to those found in many land plants. Many zygnematophytes also secrete large amounts of polysaccharide-based gels called the EPS (extracellular polymeric substance) that function in flotation and gliding, serving as platforms for establishing communication conduits of biofilms and as protection against water loss (Boney, 1981; Domozych and Domozych, 2008; Domozych *et al.*, 2009). The secretory apparatus that is required for the production of this ECM is often very extensive. In some unicellular desmids such as *Penium margaritaceum*, *Closterium acerosum* and *Micrasterias denticulate* (Lütz-Meindl, 2016; Domozych *et al.*, 2020), each cell often contains well over 100 Golgi bodies, and each Golgi body has the capability of processing multiple ECM components and packaging them in various types of vesicles for subsequent transportation to the peripheral cytoplasm. Here, the vesicles enter actin-mediated cytoplasmic streaming channels and stream around the cell periphery. Different vesicles are then selectively removed from the streaming channels and fuse with specific plasma membrane sites where they secrete their constituents to the outside of the cell. Their fast growth rates and ease in experimental manipulation and multiple microscopy-based imaging make these unicellular zygnematophytes valuable for ‘dissecting’ ECM processing pathways in late divergent charophytes (Lütz-Meindl, 2016; Domozych and Bagdan, 2022) and elucidating subcellular systems that may have been key in successful colonization of land by ancient charophyte algae. Their unicellular phenotype and ease in experimental manipulation and screening (Rydahl *et al.*, 2015) also makes these algae ideal specimens for screening potential chemical agents that target the Golgi apparatus and secretory machinery. In a screening of multiple chemical agents, we noted that endosidin 5 (ES5) results in significant alterations to Golgi apparatus structure and secretion dynamics in *Penium margaritaceum*. Multiple light- and electron microscopy-based protocols were employed and the acquired data synthesized in order to identify the subcellular targets of ES5 activity. We also examined the effects of two other endomembrane-targeting agents, brefeldin A (BFA) and concanamycin A (ConcA), and compared their effects to that of ES5.

## MATERIALS AND METHODS

### General

*Penium margaritaceum* was cultured in Woods Hole Medium supplemented with 5 % soil extract (Carolina Biological Supply, USA) using previously described methods (Rydahl *et al.*, 2015). Live cells from 7–10-d-old subcultures were harvested by centrifugation at 700 *g* for 1 min. The cell pellets were washed three times with fresh Woods Hole Medium + soil extract (WHS) and collected by centrifugation.

### Screening protocol: EPS production

For experimental cultures, 12-well uncoated tissue culture Petri dishes were used. One litre of WHS was added to each well of a 12-well uncoated tissue culture Petri dish. Various concentrations of each endosidin, BFA and ConcA were added to each well along with 0.75 µm FITC-fluorescent microspheres (Polysciences, USA). Endosidins 3, 5, 7 and 9 were purchased from ChemBridge Chemicals (USA). Endosidin 2, ConcA and BFA were purchased from Sigma Aldrich (USA). To each well 20 µL of dense cell suspension obtained from the pellet of washed cells (see above) was added. The wells were gently mixed and the dish sealed with Parafilm. Plates were cultured for 24 h under 74 µmol photons m^−2^ s^−1^ of cool white fluorescent light with a 16 : 8-h light–dark cycle. The wells were then observed with an IX-83 inverted light microscope (Olympus, USA) with FITC optics. The presence or absence of EPS trails or ensheathments were noted. For those cultures where gliding trails were not observed, the cells were harvested, centrifuged as above, washed three times with fresh WHS, resuspended in WHS containing fluorescent beads and cultured as above. Wells were observed after 24–72 h to determine if the ability to form gliding trails was recovered.

### Screening protocol: cell wall expansion

To examine cell wall expansion following treatment with the perturbation agents, cell walls were labelled with an anti-pectin antibody, JIM5, followed by a TRITC-labelled secondary antibody as previously described (Domozych *et al.*, 2014). Twenty microlitres of cell suspension of JIM5-TRITC-labelled cells was added to the wells. Cells were cultured as above for 24 h and observed with an Olympus IX-83 inverted light microscope equipped with TRITC optics. Cell wall expansion was determined by the presence of unlabelled zones at the cell isthmus, surrounded by JIM5-labelled zones (Domozych *et al.*, 2014). If no wall expansion was noted, cells were harvested from the well, centrifuged as above, washed three times with WHS and then resuspended in 1 mL of fresh WHS. The cells were cultured as above and monitored for cell expansion within 24–48 h.

An estimate of the percentage of new cell wall material produced was done by assuming the cell to be rectangular in shape. The percentage of new cell wall material was calculated by:


$$\begin{array}{l} \%\;New=\frac{\left(L_{t}-L_{o}\right)}{L_{t}}*100\% \end{array}$$

where $L_{t}$ is the total length of the cell and $L_{o}$ is the zone(s) composed of the old cell wall material that was labelled with JIM5-TRITC. The average percentage was calculated from all of the cells found in three images taken on an Olympus IX83 using the 10× objective (typically ~10 cells per image). No difference in the average/s.d. of new cell wall production was seen in control cells measured from using three images or when the number of images was increased to 10.

For time-lapse imaging of cell wall expansion, *Penium* cells walls were labelled as described above. Labelled cells were kept in the dark until use. Cells were immobilized in 2 % agarose solution within the Petri dish, and covered with fresh WHS. Time-lapse images were taken every hour with an Olympus IX83 inverted microscope using the 10× objective. Between images the cells were left on the microscope under white light with the power of the light set to 5 V.

### Live cell labelling

Live cells from 7–10-d-old subcultures or from wells of treated cultures were harvested by centrifugation at 800 *g* for 1 min were collected in 1.5-mL microcentrifuge tubes and cells were concentrated by centrifuging at 2500 *g* for 1 min. The cell pellet was washed three times with distilled water and resuspended in a final of volume of 1 mL. The cells were then incubated with either 1.25 μm Yeast Vacuole Membrane marker MDY-64 (Invitrogen, USA) or 2 μm LysoTracker™ Red (Invitrogen, USA), for 30 min in the dark with constant rotation, and washed three times with distilled water. Cells were then imaged on a Fluoview 1200 confocal laser scanning microscope (Olympus, USA) using either the FITC filter to image the MDY-64 label or TRITC filter to image the LysoTracker^TM^ Red label. The TOTO filter was used to view chloroplast autofluorescence.

### TEM preparation

Cells were harvested from 7–14-d-old cultures and washed three times with WHS as described above. The cell pellet was resuspended in 0.5 mL of WHS. Cells were then spray frozen using a commercial artist’s airbrush into 10 mL of liquid propane cooled to −185° C in a dewar of liquid nitrogen. The frozen cells were poured into precooled (−80° C) glass scintillation vials containing 0.5 % glutaraldehyde/0.2 % tannic acid in acetone (for osmicated ultrastructural analysis) The vials were placed in a −80° C freezer for 24 h. For cells prepared for osmicated ultrastructural analysis, 0.1 g of osmium tetroxide was added to the scintillation vial and the vial was placed back in the −80° C freezer for 24 h. After this time, the vial was slowly warmed to room temperature over 16 h. The cells were then collected into a pellet by centrifugation at 700 *g* for 1 min. The supernatant was discarded and the pellet was washed with acetone and recentrifuged. This was repeated twice more. The cells/pellet was then infiltrated for 3 h each in cocktails of 25 % Spurrs Low Viscosity Plastic (SLVP; EMS, USA)/75 % acetone, 50 % SLVP/50 % acetone and 75 % SLVP/25 % acetone at room temperature (RT). The cells were then placed in 100 % SLVP for 2 h at RT. The cells were then pelleted into Beem capsules (EMS, USA) and polymerized at 55° C for 8 h.

For ultrastructural analysis, 70–100-nm sections were cut using a Diatome diamond knife with a LeicaUltracut microtome, collected on Formvar-coated copper grids (EMS, USA) and stained with conventional uranyless/lead citrate (EMS, USA). Cells were viewed with an Hitachi 5800 TEM or a Zeiss Libra TEM at 120 kV.

### Tomography

For tomography, serial sections of osmicated cells embedded in SLVP were collected on copper slot grids coated with Formvar. Serial sections (150 nm) were viewed on a Libra 120 plus TEM (Zeiss, Germany) or HT7800 TEM (Hitatchi, Japan) at 120 kV from 8000 to 12 000× magnification. A tilt series of images was collected for each of the —three to six serial sections from −55° to 55° or −40° to +40° in 1° increments. Images were captured on a Cantega G2 digital camera (Olympus, USA) for images taken on the Zeiss Libra 120 plus TEM or BioSprint16 High-Definition CCD Camera (AMT, USA) for images taken on the Hitachi 7800 TEM. To obtain data for a dual axis tomogram, an orthogonal tilt series was taken after manually rotating the sample grid 90° in the sample rod. Image focus, tilt, alignment and capture were controlled by the WinTEM software (Zeiss, Germany) on the Zeiss Libra 120 plus TEM and Hitachi TEM system software (Hitachi, Japan) on the Hitachi HT7800 TEM. This process was repeated for all serial sections for each sample.

Dual axis tomograms were reconstructed using the Etomo software interface in the IMOD software package (https://bio3d.colorado.edu/imod/). The two orthogonal single-axis tomograms were merged into one with a warping procedure (Mastronarde, 1997). The above process was repeated for each section. The sections were then joined by manual alignment using Midas in the IMOD software. The alignment was then refined using fiducial markers through the adjacent tomograms (Mastronarde *et al.*, 1992).

After completing the tomographic reconstruction, the 3D model was built using the 3dmod software interface. Each cisterna was modelled as a separate object, by tracing the structures as contours in consecutive tomogram slices. After the contours were drawn on each of the tomogram slices, the IMOD mesh tool was used to create a 3D surface for each object. Vesicles around the Golgi stack were modelled by a single sphere at that position. Due to the electron beam causing some degree of section collapse, the *z*-dimension of the models was stretched by a scaling factor to accurately display the model, based on the know thickness of the sections (150 nm).

## RESULTS

**Fig. 1.**
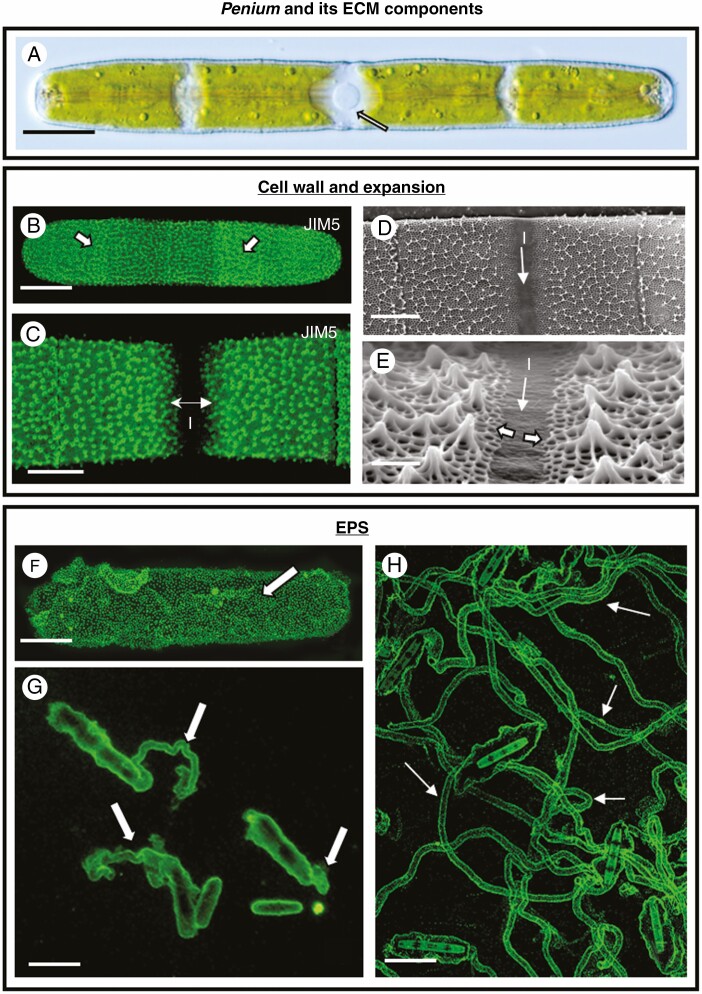
ECM production. (A) Differential interference contrast (DIC) image of *Penium*. The cell centre or isthmus (arrow) contains the nucleus and is the site of cell wall and cell expansion. Bar, 15 µm. (B) CLSM imaging of JIM5-TRITC labelling of the HG lattice (arrows) of the outer layer of the cell wall. Bar, 18 µm. (C) During cell wall expansion new HG is secreted at the isthmus (I) and displaces the older JIM5-labelled cell wall outward toward the poles (double arrow). CLSM image. Bar, 8 µm. (D, E) Scanning electron microscopy (SEM) micrographs of the cell wall near the isthmus (I). (F) Fluorescent bead labelling of the EPS surrounding a washed cell that was cultured for 2 h. The EPS forms a sheath around the cell (arrow). Bar, 13 µm. (G) Fluorescent bead labelling of the EPS now being secreted in narrow trails (arrows) in cells cultured for 4 h. Bar, 17 µm. (H) Fluorescent bead labelling of the distinct gliding trails (arrows) secreted in cells cultured overnight. Bar, 35 µm.

*Penium margaritaceum* is a unicellular, freshwater zygnematophyte with a cylindrical phenotype measuring ~17 µm in width and 125–225 µm in length (Domozych *et al.*, 2020). Each cell is composed of two semi-cells surrounding a central, isthmus zone, which is the focal zone for cell wall expansion and cell division. The nucleus resides within the isthmus zone, flanked by one or two chloroplasts in each semi-cell (Fig. 1A). The ECM is composed of a cell wall and a gel-like component known as EPS that is secreted beyond the cell wall.

The cell wall of *Penium* consists of an inner layer composed of cellulose microfibrils which act as a scaffold for the outer lattice composed of pectic polymers – primarily homogalacturonan (HG), cross-linked by calcium (Ca^2+^) ions (Domozych *et al.*, 2014). The monoclonal antibody, JIM5, labels the HG lattice that is located on the outer layer of the cell wall (Fig. 1B) and has been used as a live cell label for monitoring expansion over time (Rydahl *et al.*, 2015). During cell expansion of JIM5-labelled cells, new HG that is unlabelled (i.e. a non-fluorescent zone) is secreted at the isthmus and displaces the older labelled cell wall to the polar zones (Fig. 1C).

Secretion of the gel-like EPS can be monitored with 0.75-µm fluorescent microspheres (Polysciences, USA) which bind to these polysaccharide-based gels (Jiao *et al.*, 2020). Regions where these beads are concentrated can be detected by FLM. EPS secretion was monitored by placing washed cells (i.e. washed free of existing EPS) into a well of a 12-well uncoated culture plate containing 1 mL of growth medium with a particular agent and the fluorescent microspheres. Large numbers of treatments and concentrations of the chemical agent were then monitored using FLM. In control cells after 1 h, the fluorescent beads adhered to a thin layer of EPS secreted around the cell periphery (Fig. 1D). After 2 h, the cells secrete EPS from localized points on the cell surface (Fig. 1E) to form trails for gliding. *Penium* employs localized EPS secretion and subsequent hygroscopic swelling of the EPS as the motive force to move cells for the gliding mechanism (Domozych *et al.*, 2005). After 24 h, the trails of these gliding cells elongate substantially (Fig. 1F).

### EPS screen

The first level of screening using the fluorescent beads assessed changes to EPS secretion in treated cells and in cells recovering from chemical treatment. In the latter, treated cells that exhibited no or notably altered EPS secretion were extensively washed, placed into fresh growth medium with the fluorescent beads, cultured and observed after a 24 h period using FLM. An agent and specific treatment application were considered reversible if washed cells subsequently produced EPS trails. We selected those treatments that (1) altered EPS production, (2) did not kill cells and (3) whose effects were readily reversed by removal and recovery of cells in growth medium.

**Fig. 2.**
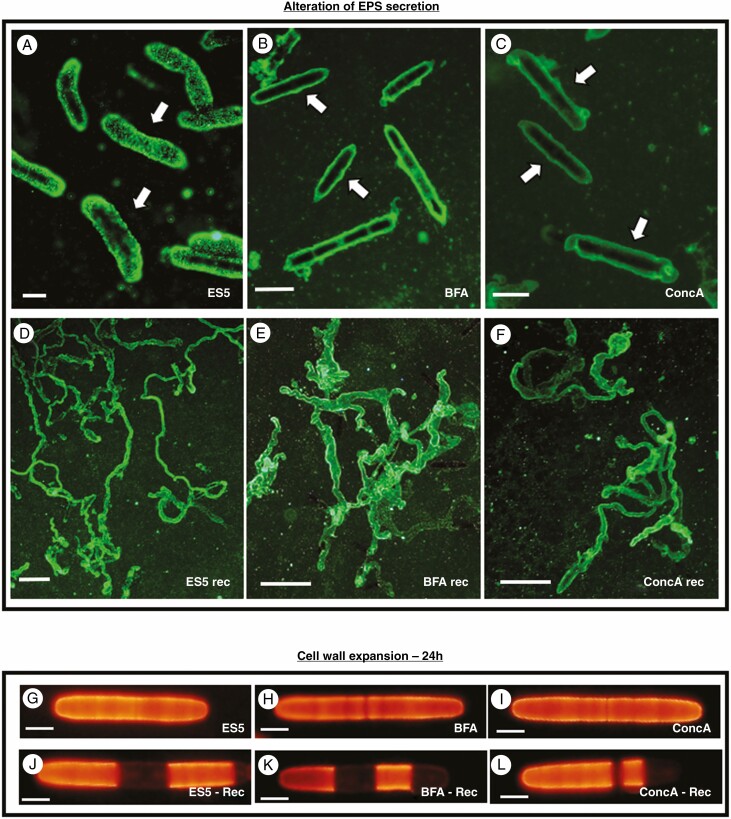
Treatment effects on EPS production. (A) EPS secretion in cells treated with ES5 overnight. EPS ensheaths each cell (arrows) but no gliding trails are formed. Bar, 17 µm. (B, C) EPS secretion in cells treated with BFA (B) or concanamycin (C) overnight. EPS ensheaths each cell (arrows) but no gliding trails are formed. Bars, 35 µm. (D–F) Recovery of EPS trails following removal of ES5 (D), BFA (E) or concanamycin A (F). Bars, 250 µm. Cell wall expansion of JIM5-TRITC labelled cells after 24 h of growth. (A, C, E) Inhibition of cell wall expansion after 24 h of treatment with ES5 (A), BFA (C) or ConcA (D). (B, D, F) Recovery of cell wall expansion after removal of inhibitory compound for 24 h. ES5 recovery (B), BFA recovery (D) and concanamycin recovery (F). Bars, 25 µm.

ES5 clearly inhibited the secretion of EPS. EPS is seen to encapsulate the cells, but no EPS trails are formed (Fig. 2A). EPS production could be restored by washing the cells with fresh media and allowing the cells to recover for 1–2 d (Fig. 2D). Similar suppression of EPS secretion (Fig. 2B, C) and recovery (Fig. 2E, F) could be seen for BFA and ConcA respectively. The final concentrations of each perturbation agent used were those which produced an effect on EPS production without killing the cells, and allowed for complete recovery of EPS production upon removal of the perturbation agent, indicating reversibility of the treatment. Concentrations were 10 µm for ES5, 1.5 µm for BFA and 1 µm for ConcA.

Endosidin 2 (ES2, 20 µm) and endosidin 3 (ES3, 15 µm) also inhibited the secretion of EPS, whereas endosidin 7 (ES7) and endosidin 9 (ES9) did not have any effect on EPS production. The concentration threshold was analysed based on the solubility of each agent and the solvent used for stock cultures (i.e. DMSO). Analysis of cells treated with DMSO up to 30 µL mL^–1^ caused no changes to EPS production (Supplementary Data Fig. S1).

### Cell wall expansion screen

The second level of screening entailed labelling live cells using JIM5 and a secondary antibody conjugated with TRITC and then placing the cells back into growth medium containing the various test chemical agents (Domozych *et al.*, 2014). Time-lapse imaging can be used to follow the bi-directional growth of the cell wall at the isthmus of each cell. Initially, a single dark zone forms at the centre of the cell which expands over time as new cell wall material is secreted at the isthmus. Following cell division, secretion of new cell wall pectins occurs at the isthmus of each daughter cell, leading to the formation of a second dark region within each cell (Supplementary Data Fig. S2A–J).

For cell wall expansion screening, JIM5-TRTIC-labelled cells were checked for unlabelled cell wall regions after 24 h of treatment. Untreated cells can be seen to have expanded significantly over this time period, leading to large expanses of unlabelled cell wall (Supplementary Data Fig. S2K). Treatment with ES5, BFA and ConcA resulted in cells that did not show any significant cell wall expansion within 24 h (Fig. 2G–I). If ES5-, BFA- and ConcA-treated cells were washed with media to remove the perturbation agent and allowed to grow for an additional 1–2 d, recovery of cell wall expansion can be seen by the appearance of new unlabelled zones in the cell wall. This indicates that the action of these chemicals was reversible.

Treatment of the cells with ES2, ES3, ES7 or ES9 did not completely inhibit cell wall expansion over 24 h. When the percentage of new cell wall material was compared between control and treated cells, no difference was seen for ES2, ES7 and ES9. Treatment with ES3 (15 µm) led to a small but significant reduction in the percentage of new wall material produced in 24 h from 46 ± 6 to 34 ± 6 % (Supplementary Data Fig. S2K–P).

### Alterations in endomembrane components

**Fig. 3.**
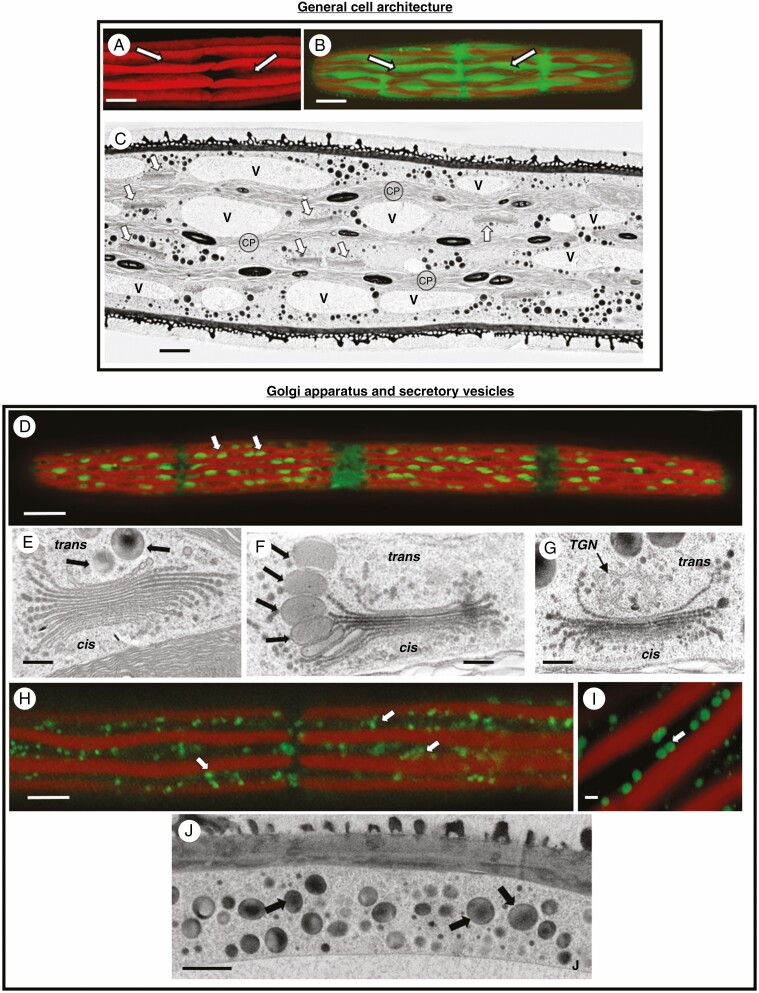
Subcellular architecture and the Golgi apparatus. (A) CLSM image of the multi-lobed chloroplast and the valleys of cytoplasm (arrows) found between each lobe. Bar, 8 µm. (B) CLSM image of a CFDA-labelled cell highlighting the cytoplasmic valleys (arrows, green) found between the lobes of the chloroplast (red). Bar, 8 µm. (C) TEM image of a longitudinal section of the cell. Linear arrays of Golgi bodies (arrows) are interspersed with the vacuolar network (V) that is positioned in lobes of cytoplasm defined by the lobes of the chloroplast (CP). Bar, 1.5 µm. (D) MDY-64 labelling of the Golgi bodies (arrows) positioned in linear arrays (arrows). CLSM images. Bar, 17 µm. (E) TEM image of a typical Golgi body. Each Golgi body consists of a stack of 12–15 cisternae. A *cis* region (*cis*) faces the chloroplast (CP) and the *trans* face (*trans*) is where vesicles emerge (arrows). Bar, 400 nm. (F) TEM image of the formation of EPS vesicles (arrows) on the Golgi body. Swellings appear at the periphery of medial to *trans* cisternae (arrows). These will bleb off the cisternae and become EPS-carrying vesicles. The *cis* (*cis*) and *trans* (*trans*) faces are also noted. Bar, 400 nm. (G) *Trans* Golgi network (TGN) found at the *trans* face (*trans*) of the Golgi body. The *cis* (*cis*) and *trans* (*trans*) faces are also noted. Bar. 450 nm. (H) CLSM image of an untreated Lysotracker-labelled cell. The fluorescent entities (arrows) are located in the peripheral cytoplasmic network. Bar, 9 µm. (I) Magnified view of Lysotracker-labelled cell. The fluorescent entities range in size from 300 to 500 nm and are probably EPS-carrying vesicles. Bar, 750 nm. (J) TEM view of the peripheral cytoplasm showing the secretory vesicles (arrows) that move in this region via cytoplasmic streaming. Bar, 1 µm.

The endomembrane system and structural alterations resulting from treatment with chemical agents were assessed by both LM and TEM methodologies. The lobed chloroplasts are the largest organelles in the cell (Fig. 3A). The lobes surround valleys of cytoplasm that extend into the interior of the cell (Fig. 3B). The cytoplasmic valleys hold mitochondria, ER, linear arrays of Golgi bodies and numerous vacuoles (Fig. 3C; see also Domozych *et al.*, 2020). Our main focus of chemical screening was the Golgi apparatus. The fluorescent label, MDY-64, was used for identification of the Golgi bodies and their orientation in the cytoplasm (Fig. 3D). In control cells, Golgi bodies are arranged in a linear orientation in the cytoplasmic valleys, appear as curved bodies and typically number 125–150 per cell. Unlike plant cells whose secretory networks have been studied in detail (e.g. *Arabidopsis*; Robinson, 2020), *Penium*’s Golgi bodies are non-mobile. Each Golgi body consists of 12–15 cisternae that are tightly stacked (Fig. 3E). Numerous vesicles are produced by the Golgi body. During EPS production, large EPS-containing vesicles form at the swollen peripheries of the medial-*trans* cisternae (Fig. 3F) and emerge from the *trans* face and not the TGN. Distinct *cis* and *trans* faces along with a TGN are also apparent (Fig. 3G). Smaller vesicles carry cell wall cargo from the Golgi body to the cell surface (see Domozych *et al.*, 2020). The EPS vesicles are carried to the peripheral cytoplasm (Fig. 3H–J) where they are transported around the cell via cytoplasmic streaming.

### Alterations in the Golgi apparatus

**Fig. 4.**
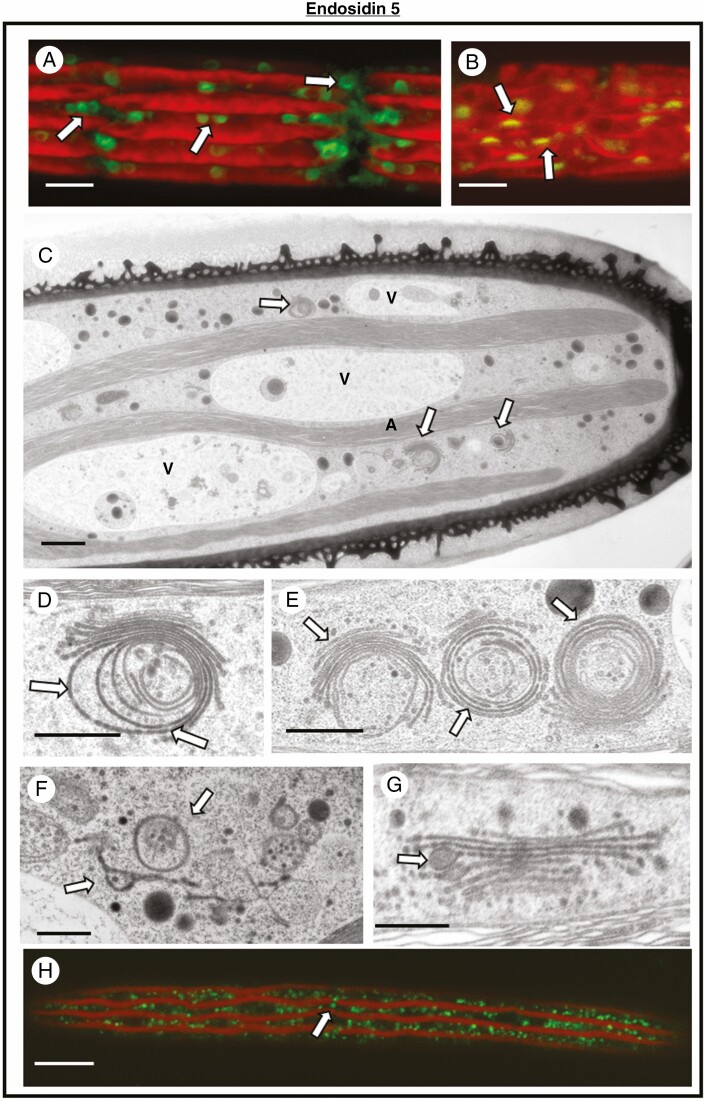
Effects of ES5 upon the Golgi apparatus. (A) MDY-64 labelling of a cell treated with ES5 overnight. The Golgi bodies (arrows) are unevenly dispersed in the cytoplasm and take on a circular profile. Bar, 5 µm. (B) MDY-64 labelling of a cell that has recovered from ES5 treatment for 24 h. The Golgi bodies (arrows) have begun to return to their original shape and cellular distribution. (C) TEM image of a cell treated with ES5 for 4 h. Note the irregular Golgi bodies (arrows) in the cytoplasmic valleys. Large vacuoles are also present. Bar, 2 µm. (D) Magnified TEM image of a Golgi body from a cell treated with ES5 for 4 h. Note the absence of vesicles and the incurling of the *trans* face cisternae (arrow). Bar, 500 nm. (E) Magnified image of a cluster of Golgi bodies (arrows). Bar, 500 nm. (F) TEM image of ES5-treated Golgi bodies after 24 h. Note that the Golgi are reduced to irregular (arrows). Bar, 500 nm. (G) Golgi body from a cell that has recoveed for 24 h after application of ES5. Note the Golgi stacking has appeared. Bar, 500 nm. (H) CLSM image of an ES5-treated Lysotracker-labelled cell. The number of vesicles is approximately the same as those found in untreated cells. Bar, 15 µm.

**Fig. 5.**
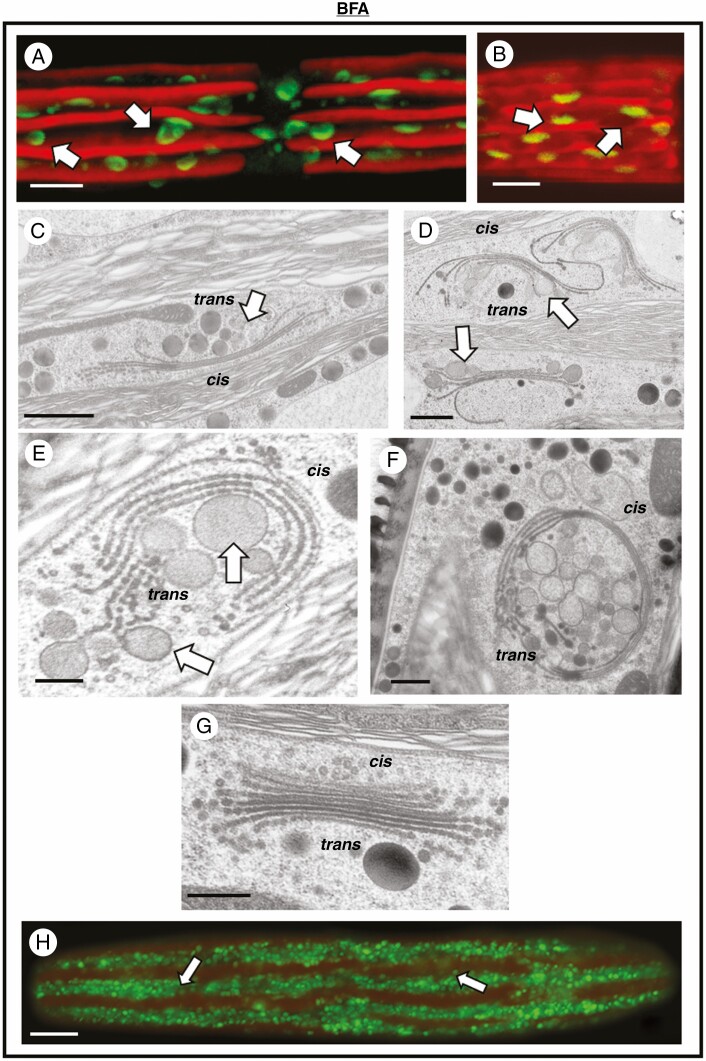
Effects of BFA on the Golgi apparatus. (A) MDY-64 labelling of a cell treated with BFA overnight. The Golgi bodies become more dispersed in the cytoplasm (arrows) and begin to change into circular entities. Bar, 5 µm. (B) MDY-64 labelling of a cell that has recovered from BFA treatment for 24 h. The Golgi bodies (arrows) have begun to return to their original shape and cellular distribution. Bar, 5 µm. (C) TEM image of Golgi body (arrow) from a cell treated for 2 h with BFA. The number cisternae per stack decreases and cisternae elongate considerably. Secretory vesicles are apparent at the *trans* face (*trans*). Bar, 500 nm. (D) Magnified view of Golgi bodies (arrows) treated with BFA for 4 h. The cisternae elongate and incurl at the *trans* face (*trans*). Bar, 500 nm. (E, F) TEM images of a Golgi body treated with BFA for 24 h. The Golgi body consists of a small stack of curled cisternae and secretory vesicles (arrows) at the *trans* face (*trans*). Bars, 250 and 500 nm respectively. (G) Reappearance of the Golgi body stack in a cell allowed to recover for 24 h. The *cis* (*cis*) and *trans* (*trans*) faces are apparent. Bar, 400 nm. (H) CLSM image of a BFA-treated, Lysotracker-labelled cell. Note a substantial increase in vesicles (arrows) in the peripheral cytoplasm. Bar, 5 µm.

ES5 produced the most striking alterations to the endomembrane system. After overnight treatment, the positioning and structure of the Golgi bodies in the cell are altered. MDY-64 labelling shows that the Golgi bodies take on a circular profile and are unevenly distributed in the cytoplasm (Fig. 4A). After thorough washing to remove ES5 and allowing the cells to recover for 24 h, the shape and distribution of the Golgi bodies return to normal (Fig. 4B). TEM imaging further highlights the structural changes to the Golgi body and its distribution in the cytoplasm (Fig. 4C). Four hours of treatment with ES5 reveal that the cisternae at the medial-to-*trans* loci of the Golgi curve inward to form circular profiles. The number of cisternae is also reduced (Fig. 4D). Aggregates of altered Golgi bodies appear in the cytoplasm and are interspersed with cytoplasm zones that are Golgi-free (Fig. 4C, E). After 24 h of treatment the Golgi body is reduced to a few scattered cisternae dispersed in the cytoplasm. After 24 h of recovery, Golgi bodies return to their original state (Fig. 5G).

Treatment with BFA produced significant changes to the endomembrane system. MDY-64 labelling of cells after overnight treatment with BFA shows that the positioning and architecture of the Golgi bodies are altered. The Golgi adopt a curved or disc-like shape and are no longer arranged in linear arrays along the cytoplasmic valleys (Fig. 5A). After washing to remove BFA and allowing the cells to recover for 24 h, the shape and distribution of Golgi bodies can be seen to return to normal (Fig. 5B). TEM imaging shows the progression of Golgi disruption over time. After 2 h of treatment, there is a reduction in the number of cisternae and an elongation of the remaining cisternae (Fig. 5). Secretory vesicles can still be seen at the *trans* face. After 4 h of treatment, production of vesicles can still be seen, and the elongate cisternae begin to curl inward at the *trans* face (Fig. 5D). After 24 h treatment, the Golgi bodies have curled inward even further, surrounding the secretory vesicles (Fig. 5E).

**Fig. 6.**
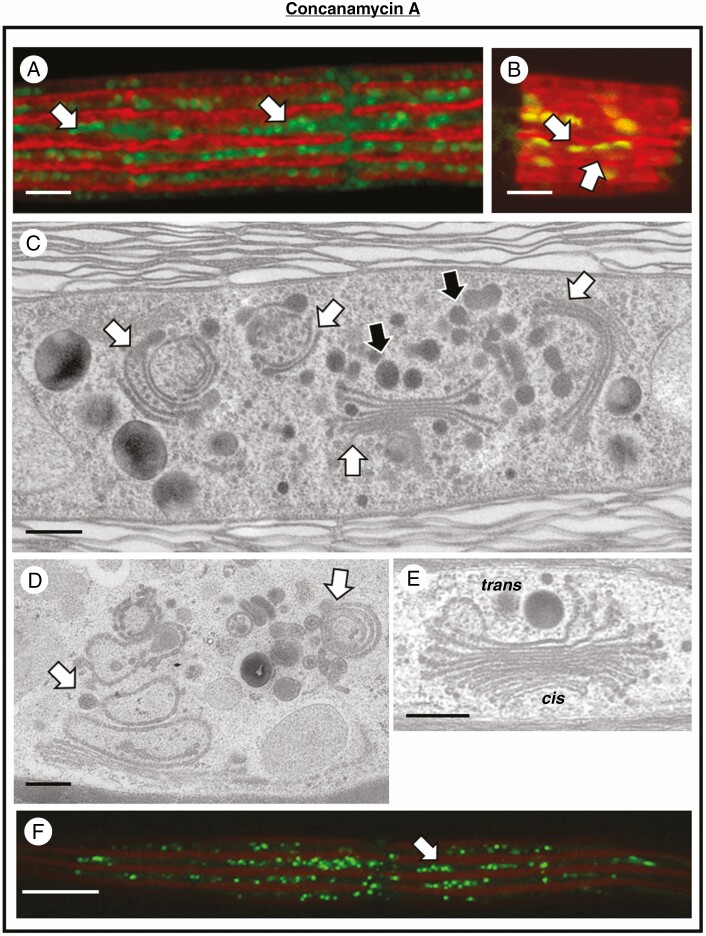
Effects of ConcA on the Golgi apparatus. (A) MDY-64 labelling of a cell treated with ConcA overnight. A large number of small, round Golgi bodies are found in the cytoplasm (arrows) and are often found in closely packed linear arrays. Bar, 5 µm. (B) MDY-64 labelling of a cell that has recovered from ConcA treatment for 24 h. The Golgi bodies (arrows) have begun to return to their original shape and cellular distribution. Bar, 5 µm. (C) TEM image of Golgi bodies from a cell treated with concanamycin for 4 h. The number of cisternae per Golgi stack decreases (white arrows) and Golgi bodies aggregate in the cytoplasm. Secretory vesicles are still produced (black arrows). Bar, 500 nm. (D) Altered Golgi bodies in a cell treated with concanamycin for 24 h. Each Golgi body (arrows) is reduced to a few cisternae. MVBs are formed (black arrows). Bar, 400 nm. (E) The typical cisternal stack returns after recovery for 24 h from concanamycin. The *cis* and *trans* faces are also apparent. Bar, 400 nm. (F) Lysotracker labelling of a cell treated with concanamycin for 24 h. The number of vesicles is approximately the same as those found in untreated cells. Bar, 15 µm.

ConcA treatment also resulted in substantial changes to the endomembrane system. After overnight treatment with concanamycin, MDY-64 labelling shows a large number of small, round Golgi bodies that are often clustered together (Fig. 6A). After recovery for 24 h, the shape, number and distribution of Golgi bodies returns to normal (Fig. 6B). TEM imaging of cells treated for 4 h with ConcA shows a reduction in the number of cisternae in Golgi bodies. The Golgi bodies form aggregates in the cell. In some Golgi bodies, the *trans* face cisternae curve inward. Swelling can often be seen at the ends of the cisternae, as well as an accumulation of vesicles near these Golgi bodies (Fig. 6C). After 24 h of treatment, the Golgi bodies further transform into irregular cisternal aggregates, including the formation of multivesicular bodies (MVBs) (Fig. 6D). Golgi bodies return to their normal state after 24 h of recovery (Fig. 6E).

ES7 and ES9 did not alter EPS production or cell wall expansion and so were not analysed for structural changes to the Golgi apparatus. ES2 and ES3 were assessed for changes to the Golgi apparatus. No structural changes were seen for ES2-treated cells, where ES3 treatment led to a mixture of normal and altered Golgi (Supplementary Data Fig. S3).

### Alterations in the vesicle network

In ES5-treated cells, labelling with lysotracker shows vesicles in the peripheral cytoplasm in similar numbers to that seen in untreated cells (Fig. 4H), in agreement with the number of vesicles seen in the TEM micrographs of ES5-treated cells (Fig. 4C). Cells treated with ConcA also show a similar number of vesicles in the peripheral cytoplasm to that observed in untreated cells using lysotracker fluorescence (Fig. 6F). BFA-treated cells, on the other hand, display a large increase in the number of vesicles seen in the cytoplasm using lysotracker (Fig. 5H) and in TEM images (Fig. 5C).

### Alterations in the pectin lattice

We used JIM5 labelling and freeze shattering/SEM protocols to monitor changes to the outer wall, pectin-based lattice in treated cells. ES5 and ConcA treatments resulted in a decrease or absence of the HG lattice while no change was observed under BFA treatment. Based on the cell expansion experiment, we posit that BFA stopped the secretion and organization of the cell wall. ES5 and ConcA allowed some cell expansion but the HG lattice was incompletely formed. In all cases, the production of the lattice returned to normal upon recovery.

### Tomography models of Golgi bodies

**Fig. 7.**
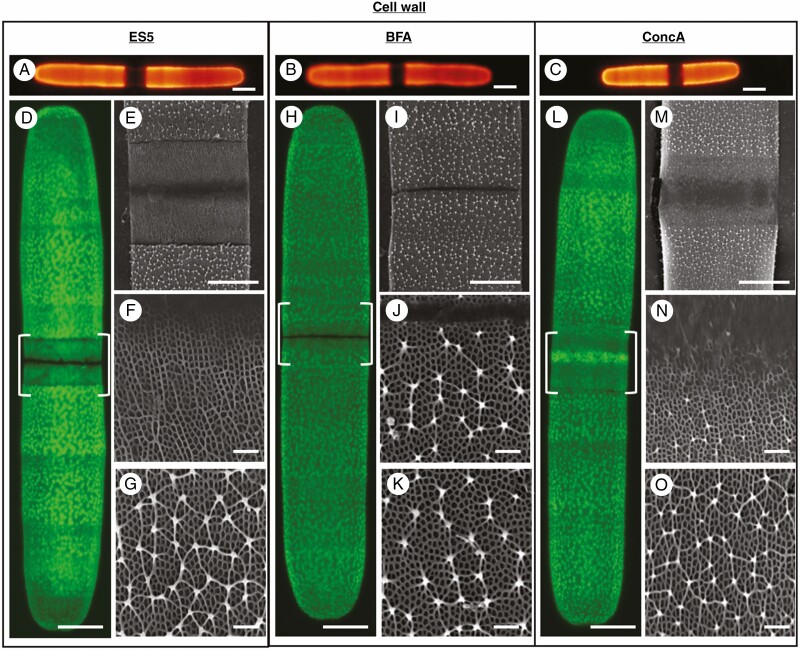
Changes to cell wall pectin structure following 48 h of treatment. (A–C) Slight expansion in cell wall seen in JIM5-FITC-labelled cells after 48 h of treatment with ES5 (A), BFA (B) and ConcA (C). Bars, 20 µm. (D) Labelling of cell wall with anti-pectin antibody, JIM5, following 48 h of treatment with ES5. Alteration of pectin lattice of newly synthesized cell wall near the isthmus zone is indicated with brackets. Bar, 10 µm. (E–G) Scanning electron micrographs of cell wall pectin of ES5-treated cells. (E) SEM micrograph of isthmus and surrounding cell wall. Bar, 5 µm. (F) Cell wall material immediately adjacent to the isthmus. Bar, 1 µm. (G) Cell wall material far from the isthmus. Bar, 1 µm. (H) Labelling of cell wall with anti-pectin antibody, JIM5, following 48 h of treatment with BFA. Pectin lattice near the isthmus zone is indicated with brackets. Bar, 10 µm. (I–K) Scanning electron micrographs of cell wall pectin of BFA-treated cells. (I) SEM micrograph of isthmus and surrounding cell wall. Bar, 5 µm. (J) Cell wall material immediately adjacent to the isthmus. Bar, 1 µm. (K) Cell wall material far from the isthmus. Bar, 1 µm. (L) Labelling of cell wall with anti-pectin antibody, JIM5, following 48 h of treatment with ConcA. Alteration of pectin lattice of newly synthesized cell wall near the isthmus zone is indicated with brackets. Bar, 10 µm. (M–O) Scanning electron micrographs of cell wall pectin of ConcA-treated cells. (M) SEM micrograph of isthmus and surrounding cell wall. Bar, 5 µm. (N) Cell wall material immediately adjacent to the isthmus. Bar, 1 µm. (O) Cell wall material far from the isthmus. Bar, 1 µm.

To acquire additional structural information about the Golgi bodies, dual-axis tomography of serial EM sections was performed. The serial tomograms were then joined to achieve a total sample thickness of 450–900 nm. Three-dimensional profiles could be reconstructed from the tomography data using the 3dmod software (Kremer *et al.*, 1996). The differences between the Golgi of treated and untreated cells becomes even more obvious in the reconstructions. Control Golgi bodies consist of relatively flat stacks of membranes, with clearly distinguishable *cis*, medial and *trans* regions. Small vesicles can be seen at both the *cis* and *trans* faces, and many large secretory vesicles can be seen at the *trans* face (Fig. 7A–C, and Supplementary Data Video S1]. The Golgi of ES5 treated cells becomes altered in several distinct ways (Fig. 7D, and Data S2). The number of cisternae is greatly reduced. While the cisternae at the *cis* face remain relatively flat, the two *trans*-most cisternae form cup-like structures, which are beginning to fuse at the upper edge. Small vesicles can be seen surrounding the Golgi body. Some secretory vesicles are present near the *trans* face but are reduced in number compared to the control. The Golgi of BFA-treated bodies also show interesting features in the 3D reconstruction (Fig. 7E, and Supplementary Data Video S3). The elongated cisternae are curved at the centre but the edges of the cisternae remain flat, creating a ‘U’-like shape. While substantial curvature is seen, no cup-like structures or fusion between cisternae was seen. Lastly, tomographic reconstruction was performed on Golgi of ConcA-treated cells (Fig. 7F, and Video S4). Several Golgi bodies can be seen in close proximity. The cisternae can be seen to have a variety of different curved shapes including hollow tubes and cup-like structures. Some of the internal-most membranes have fused completely at both the top and bottom to form spheres surrounding smaller vesicles, forming MVBs. Secretory vesicles are still seen surrounding the Golgi bodies. A tomographic reconstruction of a curved Golgi body found in an ES3-treated cell was also (Fig. S4 and Video S5).

**Fig. 8.**
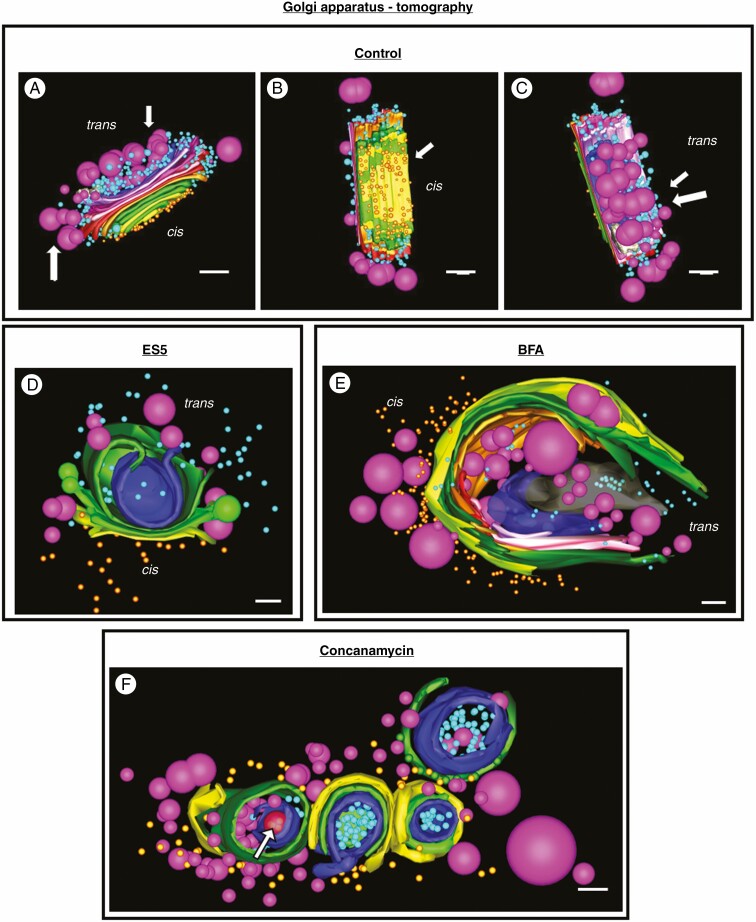
Electron tomography of the Golgi body in treated and untreated cells. (A–C) 3D model of control Golgi. The stacks of cisternae are relatively flat, and the *cis* and *trans* faces are readily identifiable. The Golgi body is surrounded by small vesicles at both the *cis* (orange) and *trans* (cyan) faces. Large secretory vesicles (magenta) can be found only at the *trans* face. Bars, 250 nm. (D) 3D model of Golgi body after ES5 treatment. The number of cisternae is reduced compared to the control. Cisternae at the *cis* face remain relatively flat, whereas cisternae at the *trans* face curve inward and form cup-like structures. The Golgi body is surrounded by small vesicles at both the *cis* (orange) and *trans* (cyan) faces. Large secretory vesicles (magenta) are found at the *trans* face. Bar, 200 nm. (E) 3D model of Golgi body after BFA treatment. The number of cisternae is reduced compared to the control, and cisternae become elongated. The cisternae are relatively straight at the edges but curve tightly near their midpoint, towards the *trans* face, forming a ‘U’ like structure. The Golgi body is surrounded by small vesicles at both the *cis* (orange) and *trans* (cyan) faces. Large secretory vesicles (magenta) can be found at both the *cis* and *trans* faces, as well as caught between cisternae. Bar, 200 nm. (F) 3D model of Golgi bodies after concanamycin treatment. Several Golgi bodies can be found in close proximity. The number and size of cisternae is reduced, and the cisternae are curved, often forming cup- or tube-like structures. The Golgi body is surrounded by small vesicles at the outer (*cis*) face (orange) and filled with small vesicles (cyan) within the inner (*trans*) face. In some cases, multi-vesicular bodies (arrow) are formed. Many secretory vesicles (magenta) can be seen surrounding the Golgi bodies. Bar, 200 nm.

Tomography data confirm structural information about the Golgi bodies seen in the micrographs obtained from thin TEM sections and also provide unique insights into the Golgi structure, particularly for treated samples. The 3D curling of the cisternae in ES5-, BFA- and ConcA-treated cells can only be elucidated from tomographic data (Fig. 8).

## DISCUSSION

A multifunctional ECM that dynamically modulates in response to environmental pressures was critical to the evolution of plants and serves as a foundation for a plant cell’s life functions. To process the large numbers of diverse ECM components, plant cells employ a complex endomembrane system that synthesizes, packages and transports ECM cargoes to the cell surface. Central to this secretory processing is the Golgi apparatus. While recent research has greatly improved our understanding of Golgi-based membrane trafficking in plants, many basic questions remain to be answered. For example, how do Golgi dynamics change in response to particular abiotic and biotic stress agents in order to produce necessary modulations in the ECM? Likewise, how has the endomembrane system evolved in ancestral taxa of plants and how might information derived from studies of charophytes help provide insight into plant evolution and ecophysiology? *Penium* is an outstanding alga for elucidating endomembrane structure/function and ECM secretion in zygnematophytes. It can also provide insights into those subcellular systems that are critical for life at the aquatic/terrestrial interface which may have been key in the successful colonization of land by an ancient alga. Some of the efficiacious attributes of *Penium* include: (1) it has a well-characterized ECM that includes a pectin-rich cell wall and extensive EPS, (2) it is both easy to maintain in the laboratory and screen using large arrays of chemical agents, (3) it lends itself well for live cell fluorescence microscopy with a variety of probes (e.g. antibodies, subcellular labelling agents) and electron microscopy (e.g. cryofixation by spray freezing), and (4) it has an extensive endomembrane system that processes the ECM (Domozych *et al.*, 2020). In this study, we exploit these attributes to analyse the effects of ES5 and other endomembrane system-disrupting agents.

Chemical biology and specifically the identification/application of chemicals that target and perturb endomembrane structure and function have become one of the valuable tools in the dissection of the endomembrane system. In this study, we describe multiple screening techniques that can be used to analyse the effects of endomembrane-targeting chemicals in the unicellular charophyte *Penium margaritaceum*. Following the initial screens, we focused our analyses on the effects of ES5, one of the low-molecular-weight, bioactive molecules that have been shown to interact with endomembrane trafficking and affect the architecture of endomembrane compartments (Robert *et al.*, 2008; Drakakaki *et al.*, 2011; Dejonghe and Russinova, 2017; Davis *et al.*, 2020; De Caroli *et al.*, 2020). We also compared these effects with BFA, a widely used endomembrane system-disrupting agent that has been used with *Penium* (Domozych *et al.*, 2020), other zygnematophytes and green algae (Dairman *et al.*, 1995; Salomon and Meindl, 1996; Domozych, 1999; Hummel *et al.*, 2007; Lütz-Meindl, 2016), and ConcA), a widely used V-ATPase inhibitor that has been shown to disrupt the endomembrane system in plants (Seidl, 2022).

ES5 is the endosidin that causes the most notable perturbations to ECM production and the endomembrane system. During the first 4 h of treatment there were substantial alterations to Golgi body positioning in the cytoplasmic valleys and the structure of the Golgi apparatus. Golgi bodies no longer aligned in the typical linear arrays and the cisternae of the medial-to-*trans* loci of the Golgi body curl inward at the *trans* face. Secretory vesicle number decreases near the Golgi bodies but they are still present in the peripheral cytoplasm. After longer treatments (e.g. 24 h), the Golgi bodies are reduced to a few scattered cisternae. During ES5 treatment, EPS and cell wall secretion are inhibited or curtailed substantially. These effects indicate that ES5 directly targets the structural integrity of the Golgi apparatus. TEM imaging revealed that the *trans*-face cisternae are the first to be disrupted, with the *cis*-face cisternae remaining relatively intact in early stages of treatment We also noted that typical TGN is not found in ES5-treated cells. It is possible that the movement of cisternae towards the *trans* face and their ultimate transformation into the TGN are inhibited, which in turn leads to curling of the cisternae. At present, little information is available concerning factors that maintain the infrastructure of the Golgi body of *Penium*. For example, this would include specific proteins, such as Golgins and Golgi Reassembly and Stacking Proteins (GRASPs; Rui *et al.*, 2022), that maintain Golgi structure, including cisternal stacking. However, the recent sequencing of the *Penium* genome has revealed many Golgin-like genes (Jiao *et al.*, 2020). Further work will be required at both the molecular and cellular levels to identify those Golgi-specific maintenance components and the exact roles of ES5 in their alteration.

ES7 and ES9 had no discernible impact on the secretion of ECM components and were not considered further for this study. Both ES2 and ES3 were found to inhibit EPS production. ES2 has been shown to inhibit exocytosis in plants and human cells and to specifically target the EXO70 subunit of the exocyst complex (Zhang *et al.*, 2016). Little is known about the exocyst of *Penium* but screening of the *Penium* genome reveals many candidate genes for the exocyst in this alga (Jiao *et al.* 2020). ES3, interestingly, showed a slight inhibition of cell wall expansion and a mixture of normal and curved Golgi structures. The curved Golgi bodies in ES3-treated cells display similarities to those following treatment with ES5 (Supplementary Data Fig. S3). ES3 has been shown to affect membrane trafficking but the specific target is unknown (Drakakaki *et al.*, 2011).

In a previous study (Domozych *et al.*, 2020), treatment of *Penium* with BFA resulted in notable changes to both the endomembrane system and cell wall expansion at 1 μg mL^–1^ (3.57 µm) after 2 h. In this study we tested lower concentrations of BFA which would not kill the cells over 24–48 h of treatment. *Penium*’s endomembrane system was shown to be very sensitive to BFA even at a very low concentration (1.0 µm). As in the previous study, BFA altered the positioning of the Golgi bodies in the cytoplasm and disrupted Golgi architecture. The number of cisternae decreases from 12–15 to fewer than six and cisternae become greatly expanded. Both EPS secretion and cell wall expansion are inhibited. Secretory vesicle production in treated cells for up to 48 h still occurs at the Golgi body and the peripheral cytoplasm is filled with secretory vesicles. None of the large vacuole-like structures found in the previous study (Domozych *et al.*, 2020) were seen here, probably due to the reduction in the concentration of BFA. These results indicate that in addition to BFA’s disruption of the Golgi architecture, this agent also significantly reduces or inhibits secretory vesicle fusion with the plasma membrane and secretion. This corresponds with other studies whereby BFA caused a near complete blockage of constitutive secretion in both animal cells (Rosa *et al.*, 1992; De Lisle and Bansai, 1996) and plant cells (Takác *et al.*, 2011; Drdová et al, 2013; Rounds *et al.*, 2014; Scali *et al.*, 2021). BFA has also been to shown to cause disassembly of the Golgi apparatus and the absorbance of most Golgi components into the ER in plant cells (Nebenführ *et al.*, 2002; Lam *et al.*, 2009; Foissner *et al.*, 2020). It was shown that the majority of Golgi cisternae fuse directly with the ER, leading to the formation of an ER–Golgi hybrid compartment. Additionally, BFA treatment often causes both the Golgi apparatus and TGN to form distinct aggregates or ‘BFA bodies’ (Lam *et al.*, 2009). In *Penium*, neither ER–Golgi compartments nor BFA bodies were observed and in fact the altered Golgi stacks were devoid of any notable connections of the Golgi body to the ER and TGN-like structures. This leads to three conclusions: (1) BFA targets Golgi body architecture in *Penium* in a notably different way than in higher plant cells and causes effects at much lower concentrations; (2) different concentrations of BFA cause different effects on the Golgi architecture but all stop the secretion of cell wall and EPS; and (3) the production of secretory vesicles in the Golgi body probably does not require transport through the TGN. Future analyses of the TGN in *Penium* and other zygnematophytes and its role as a hub for exo- and endocytosis activities will be needed to provide a more complete understanding of endomembrane dynamics in these algae. The differences seen in BFA effects on *Penium* as compared to land plants is perhaps unsurprising due to known differences in the Golgi apparatus between *Penium* and other green organisms. These differences may limit the number of parallels that can be drawn between *Penium* and land plants, although similarities do exist. For example, BFA’s suppression of cell wall expansion as observed in *Penium* has also been observed in a diverse array of plant cells (Rutten and Knuiman, 1993; Yasuhara and Shibaoka, 2000; Grebnev *et al.*, 2020; Kim *et al.*, 2021; Yan *et al.*, 2022).

ConcA is a known inhibitor of V-ATPase, an important proton pump found throughout the endomembrane system of both plants and algae (Grunow *et al.*, 1999; Seidel, 2022). In *Chlamydomonas*, ConcA treatment caused changes to autophagy/vacuoles (Couso *et al.*, 2018). Our TEM analyses of *Penium*’s vacuolar/vesicle network did not reveal major changes but future work will be required to specifically examine both autophagy and experimental manipulation of the autophagic mechanism in this alga. In *Penium*, ConcA altered the number, positioning and architecture of the Golgi bodies. The Golgi bodies become clustered together, each only containing a few, highly curved cisternae. Swelling at the ends of the cisternae and the formation of MVBs can be seen, similar to results found in *Arabidopsis* (Dettmer *et al.*, 2006). ConcA treatment in tobacco BY-2 cells also leads to a curving of the cisternae and a reduction in cisternae number but also leads to a prominent ‘vacuolation’ of the Golgi apparatus that was not seen in *Penium* (Robinson *et al.*, 2004). Secretory vesicles are still found in the cytoplasm but both EPS secretion and cell wall expansion are inhibited. This is perhaps unsurprising as the inhibition of V-ATPase in plants has been shown to inhibit exocytosis, including delivery of cell wall material (Brüx *et al.*, 2008; Luo *et al.*, 2015; De Caroli *et al.*, 2020).

Endosidins have become important tools in ‘dissecting’ the dynamics of exo- and endocytosis in plant cells (Drakakaki *et al.*, 2011). In this study we show that ES5 is a potent perturbation agent of Golgi architecture and the processing of the cell wall and EPS of *Penium*. The exact target and action of ES5 are not known but the effects clearly differ from other endomembrane inhibitors such as BFA and ConcA. Further detailed studies will be required to elucidate the mechanism of action, but this chemical offers a new tool in the arsenal of agents available to dissect Golgi architecture and membrane trafficking in late-divergent charophytes. Such inhibitors could play an important role in our understanding of the role played by the endomembrane system in the transition of an ancient charophyte ancestor on to land.

## Supplementary Material

mcad054_suppl_Supplementary_Figure_S1

mcad054_suppl_Supplementary_Figure_S2

mcad054_suppl_Supplementary_Figure_S3

mcad054_suppl_Supplementary_Figure_S4

mcad054_suppl_Supplementary_Legends

mcad054_suppl_Supplementary_Video_S1

mcad054_suppl_Supplementary_Video_S2

mcad054_suppl_Supplementary_Video_S3

mcad054_suppl_Supplementary_Video_S4

mcad054_suppl_Supplementary_Video_S5

## SUPPLEMENTARY DATA

Fig. S1: EPS screening of other endosidins. Fig. S2: Cell wall expansion of JIM5-TRITC-labelled cells. Fig. S3: Structure of Golgi apparatus following 24 h of treatment with ES2 and ES3. Fig. S4: 3D model of curved Golgi body found in an ES3-treated cell. Videos S1–5: animations of the 3D Golgi body reconstructions modelled from the tomography data of cells following (1) no treatment, (2) ES5 treatment, (3) BFA treatment, (4) ConcA treatment and (5) ES3 treatment.
