# Supplementary figures and images for: Endosidin 5 disruption of the Golgi apparatus and extracellular matrix secretion in the unicellular charophyte Penium margaritaceum
Source: Ann Bot. 2023 Apr 20;131(6):967–83. doi: 10.1093/aob/mcad054 (PMC10332397; doi:10.1093/aob/mcad054)

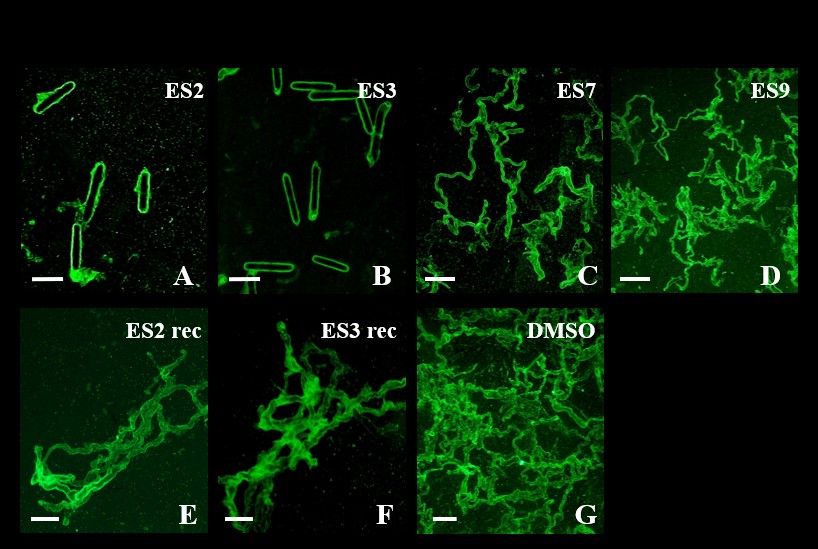

Supplement: mcad054_suppl_Supplementary_Figure_S1 [file mcad054_suppl_supplementary_figure_s1.jpeg]

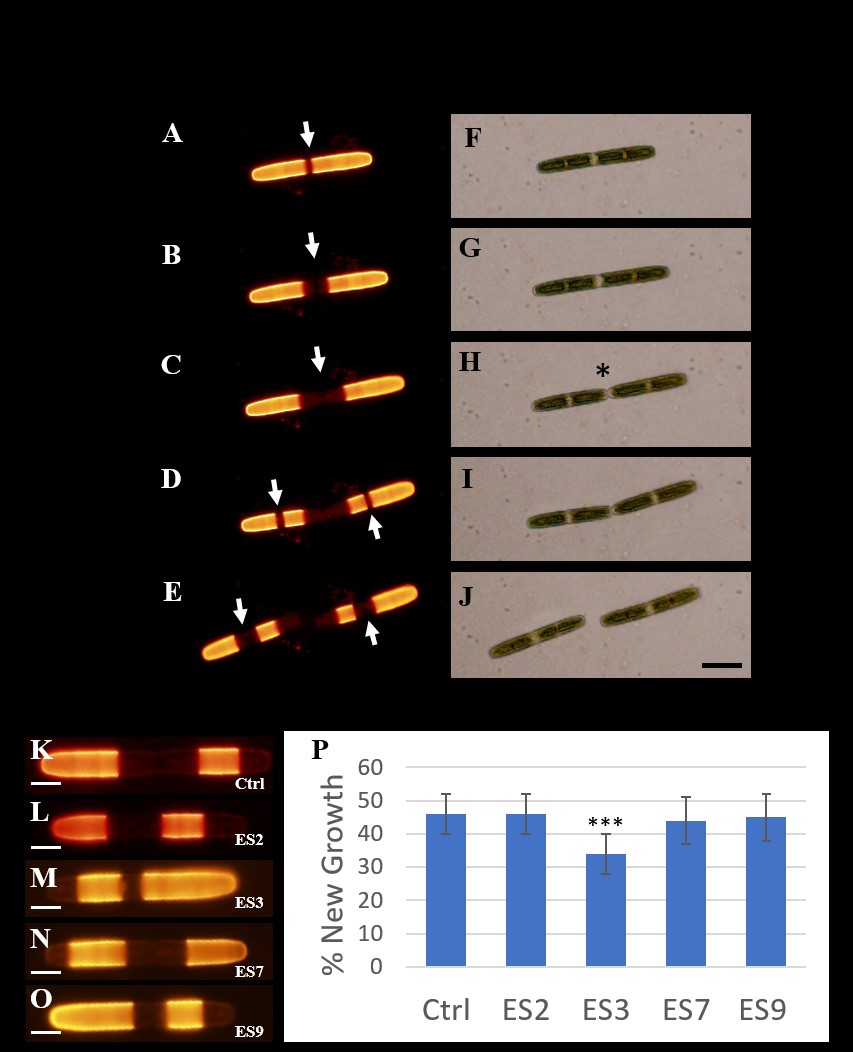

Supplement: mcad054_suppl_Supplementary_Figure_S2 [file mcad054_suppl_supplementary_figure_s2.jpeg]

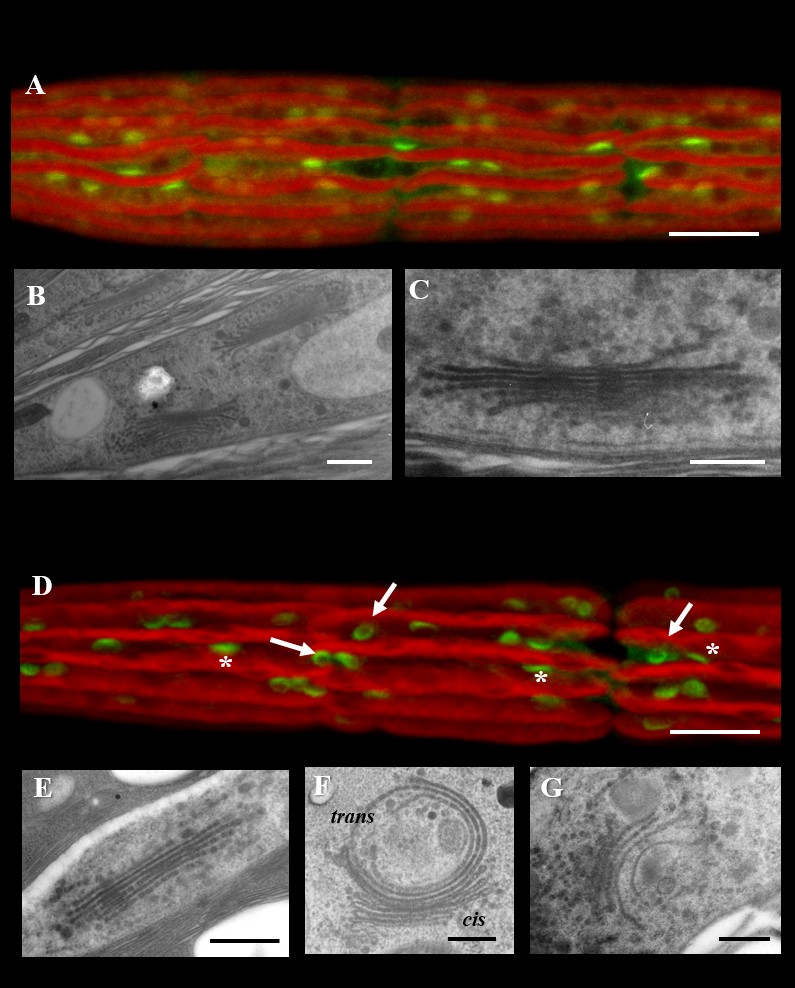

Supplement: mcad054_suppl_Supplementary_Figure_S3 [file mcad054_suppl_supplementary_figure_s3.jpeg]

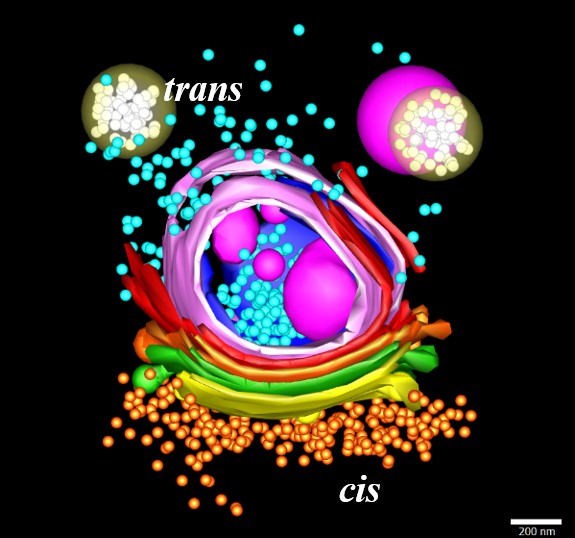

Supplement: mcad054_suppl_Supplementary_Figure_S4 [file mcad054_suppl_supplementary_figure_s4.jpeg]
