# Supplementary material for: Endosidin 5 disruption of the Golgi apparatus and extracellular matrix secretion in the unicellular charophyte Penium margaritaceum
Source: Ann Bot. 2023 Apr 20;131(6):967–83. doi: 10.1093/aob/mcad054 (PMC10332397; doi:10.1093/aob/mcad054)
Supplement: mcad054_suppl_Supplementary_Legends [file mcad054_suppl_supplementary_legends.docx]

**Supplemental Figure Legends**

Supplementary Figure 1**.** EPS screening of other Endosidins. Inhibition of EPS production following treatment with 20 µM ES2 (**A**), and 15 µM ES3 (**B**). Bars, 35 µm. EPS trails seen following treatment with 30 µM ES7 (**C**) and 30 µM ES9 (**D**). Bars, 80 µm. Recovery of EPS trail production following removal of ES2 (**E**) and ES3 (**F**). Bars, 80 µm. Formation of EPS trails following treatment with 30µL/mL DMSO (**G**). Bar, 85 µm.

Supplementary Figure 2. Cell wall expansion of JIM5-TRITC labeled cells. Time-lapse imaging of expansion of control cell (**A-J**). Expansion of unlabeled zone of the cell wall as new cell wall material is produced at the isthmus (arrow) (**A-C**). Following cell division, the daughter cells begin to expand at their isthmus zones (arrows) leading to formation of new unlabeled zones on the cell wall (**D-E**). Cell expansion viewed using brightfield imaging (**F-J**). Cell division is noted with an (*). Bar, 50 µm. Image of representative cell wall expansion of JIM5-TRITC labeled cells for control (**K**), 20 µM ES2 (**L**), 30 µM ES3 (**M**), 30µM ES7 (**N**) and 30 µM ES9 (**O**) after 24h treatment. Bar, 20 µm. Percentage of new cell wall material produced after 24h treatment (**P**). Error bars represent the standard deviation. *** Indicates p < 0.001 compared to control.

Supplementary Figure 3. Structure of Golgi Apparatus following 24 h treatment with ES2 and ES3. MDY-64 labelling of Golgi bodies following 24 h treatment with 20 µM ES2 (**A**). Golgi bodies are indistinguishable from controls. Bar, 10µm. TEM micrographs of Golgi bodies following treatment with ES2 (**B,C**). Golgi bodies are similar to control Golgi. Bars, 500 nm. MDY-64 labeling of Golgi bodies following 24 h treatment with 30 µM ES3 (**E**). A mixture of normal and altered Golgi bodies can be seen. Arrows are used to highlight some of the altered Golgi bodies and * is used to highlight normal Golgi bodies. Bar, 10 µm. TEM micrographs of Golgi bodies following treatment with ES3 with various morphologies (**E-G**). Bars, 500 nm .

Supplementary Figure 4. 3D model of curved Golgi body found in an ES3 treated cell. The stacks of cisternae are relatively flat at the *cis* face, and cisternae at the *trans* face curve inward, sometime forming a closed loop. The Golgi body is surrounded by small vesicles at both the *cis* (orange) and *trans* (cyan) faces. Large secretory vesicles (magenta) can be found at the *trans* face and in some cases trapped within the curved cisternae at the *trans* face. Bar 200 nm.

**Supplemental Videos**

Supplementary Video 1. 3D model of typical Golgi body found in *Penium*. Model was rotated around both the x and y-axes to illustrate Golgi structure.

Supplementary Video 2. 3D model of Golgi body after ES5 treatment. Model was rotated around both the x and y-axes to illustrate Golgi structure.

Supplementary Video 3. 3D model of Golgi body after BFA treatment. Model was rotated around both the x and y-axes to illustrate Golgi structure.

Supplementary Video 4. 3D model of Golgi bodies after ConcA treatment. Model was rotated around both the x and y-axes to illustrate Golgi structure.

Supplementary Video 5. 3D model of Golgi bodies after ES3 treatment. Model was rotated around both the x and y-axes to illustrate Golgi structure.
